# A Role of Kindlin-3 in Integrin αMβ2 Outside-In Signaling and the Syk-Vav1-Rac1/Cdc42 Signaling Axis

**DOI:** 10.1371/journal.pone.0056911

**Published:** 2013-02-20

**Authors:** Zhi-Hong Xue, Chen Feng, Wei-Ling Liu, Suet-Mien Tan

**Affiliations:** School of Biological Sciences, Nanyang Technological University, Singapore, Singapore; National Center for Scientific Research Demokritos, Greece

## Abstract

Integrins mediate cell-cell and cell-extracellular matrix attachments. Integrins are signaling receptors because their cytoplasmic tails are docking sites for cytoskeletal and signaling proteins. Kindlins are a family of band 4.1-ezrin-radixin-moesin-containing intracellular proteins. Apart from regulating integrin ligand-binding affinity, recent evidence suggests that kindlins are involved in integrin outside-in signaling. Kindlin-3 is expressed in platelets, hematopoietic cells and endothelial cells. In humans, loss of kindlin-3 expression accounts for the rare autosomal disease leukocyte adhesion deficiency (LAD) type III that is characterized by bleeding disorders and defective recruitment of leukocytes into sites of infection. Studies have shown that the loss of kindlin-3 expression leads to poor ligand-binding properties of β1, β2 and β3 integrin subfamilies. The leukocyte-restricted β2 integrin subfamily comprises four members, namely αLβ2, αMβ2, αXβ2 and αDβ2. Integrin αMβ2 mediates leukocyte adhesion, phagocytosis, degranulation and it is involved in the maintenance of immune tolerance. Here we provide further evidence that kindlin-3 is required for integrin αMβ2-mediated cell adhesion and spreading using transfected K562 cells that expressed endogenous kindlin-3 but not β2 integrins. K562 stable cell line expressing si-RNA targeting kindlin-3, but not control-si-RNA, and transfected with constitutively activated integrin αMβ2N329S adhered and spread poorly on iC3b. We also show that kindlin-3 is required for the integrin αMβ2-Syk-Vav1 signaling axis that regulates Rac1 and Cdc42 activities. These findings reinforce a role for kindlin-3 in integrin outside-in signaling.

## Introduction

Integrins are transmembrane receptors that mediate cell-cell and cell-extracellular matrix interactions [1]. An integrin is a heterodimer composed of an α and a β subunit. Each subunit has a large extracellular region and a transmembrane domain followed by a cytoplasmic tail [1]. The extracellular region contains ligand-binding sites whereas the cytoplasmic tail associates with intracellular proteins [2], [3]. Conformational changes in integrins are directly regulated by extracellular bivalent cations, mechanical forces, and proteins that bind integrin cytoplasmic tails [4]–[6].

Integrin αMβ2 (CD11bCD18, Mac-1, CR3) is a member of the β2 integrin subfamily [7]. Its expression is restricted to cells of the hematopoietic system and primarily that of myeloid lineage [8], [9]. It binds complement protein iC3b and a wide variety of ligands, including denatured proteins [7]. Apart from its major function as a phagocytic receptor [10]–[12], it is involved in leukocyte migration, differentiation, apoptosis, and the induction of immune tolerance [13]–[20].

In addition to the widely reported cytoskeletal proteins talins, kindlins which are also band 4.1-ezrin-radixin-moesin (FERM)-containing intracellular proteins have been shown to regulate integrin ligand-binding [21]–[24]. Kindlin-1 is epithelial-specific and kindlin-2 is widely expressed in different cell types [25]. Kindlin-3 is expressed in platelets, hematopoietic cells and endothelial cells [26], [27]. Defective kindlin-3 expression leads to LAD III that is characterized by bleeding disorders and a compromised immune system because of dysfunctional platelet αIIbβ3 and leukocyte β2 integrins, respectively [26], [28]–[32].

Kindlin has different sub-domains serving specific functions. The F0 sub-domain has been shown to target kindlin-1 to focal adhesion sites [33]. A loop in the F1 sub-domain of kindlin-1 has been shown to bind phosphatidylserine lipid headgroup [34]. A pleckstrin homology (PH) domain that is inserted into the F2 sub-domain allows kindlin-2 to bind phosphatidylinositol phosphate(s) [33], [35]–[37]. The F3 sub-domain of kindlins binds to the membrane distal NxxY/F motif in integrin β cytoplasmic tails [7], [30], [38], [39]. In addition to integrins, binding partners of kindlins that have been reported are integrin linked kinase (ILK), migfilin, receptor for activated-C kinase 1 (RACK1), and β-catenin [40]–[43].

Many studies have demonstrated a role of kindlins in inside-out activation of integrins (reviewed in [23], [38], [44]), but there is gaining evidence that kindlins are also involved in integrin outside-in signaling. In kerantinocytes, β1 integrin regulates RhoGTPase activity and it involves kindlin-1 [45]. Reduced kindlin-2 expression in osteoblasts diminished the activation of Rac1, Akt and AP-1 [46]. Platelets from kindlin-3 knockout mice showed defective spreading on fibrinogen even though integrin αIIbβ3 was activated by Mn^2+^ [26]. Kindlin-3 is not only important for integrin αLβ2 activation (inside-out) [31], it is also required for integrin αLβ2 outside-in signaling because kindlin-3 deficient LAD III EBV-transformed B lymphoblasts failed to adhere on densely coated ICAM-1 [47]. Recently, we have also shown that K562 cells with reduced kindlin-3 expression were defective in their spreading on ICAM-1 or fibrinogen despite over-expressing constitutively activated integrin αLβ2 or αIIbβ3, respectively [42].

Kindlin-3 is required for integrin αMβ2 inside-out activation in PMNs [30]. However, to our knowledge there is still little information on the role of kindlin-3 in integrin αMβ2 outside-in signaling. Herein, we show that kindlin-3 mediates integrin αMβ2 outside-in signal transduction and its involvement in integrin αMβ2-Syk-Vav1 signaling axis that regulates Rac1 and Cdc42 activities.

## Materials and Methods

### Antibodies

Function-blocking mouse mAb LPM19c (specific to integrin αM subunit) and activating mAb KIM185 (specific to integrin β2 subunit) (American Type Culture Collection, ATCC) have been previously described [48]. The mAb KIM127 (ATCC) that reports activated β2 integrins has been previously described [49], [50]. The following antibodies were purchased from different commercial sources. Mouse anti-talin (8d4) and mouse IgG (MOPC-31c) were from Sigma-Aldrich, St Louis, MO. Mouse anti-Syk antibody, mouse anti-phosphotyrosine (PY20), mouse anti-actin antibody and APC-conjugated goat anti-mouse IgG were from BD Biosciences, San Jose, CA. Rabbit anti-Syk pY525/526, rabbit anti-Vav1, and rabbit anti-Cdc42 were from Cell Signaling Technology, Danvers, MA. Mouse anti-Rac1 was from Merck Millipore, Rockland, MA. Rabbit anti-RhoA antibody and rabbit anti-PKCδ (C-20) antibody were from Santa Cruz Biotechnology, Santa Cruz, CA. Rabbit anti-GST antibody was from Delta Biolabs, Gilroy, CA. Rabbit mAb against integrin αM used in immunoblotting was from Abcam, Hong Kong. Rat mAb clone 9 against kindlin-3 was generated in our lab [42]. For ECL immunoblotting, HRP-goat anti-mouse IgG and HRP-goat anti-rabbit IgG secondary antibodies were from Avansta, CA. HRP-goat anti-rat IgG secondary antibody was from GE Healthcare, Piscataway, NJ.

### Cell adhesion assay

Static cell adhesion assay was performed essentially as previously described [48]. Briefly, each well of the Polysorb microtiter plate (Nunc, Denmark) was coated with iC3b (7.5 µg/ml) (Complement Technology, Tyler, TX) or BSA (100 µg/ml) (Sigma) in 50 mM bicarbonate buffer (pH 9.2) at 4°C overnight. Non-specific binding sites were blocked with 0.2% (w/v) polyvinylpyrrolidone (PVP) (MW 10,000) (Sigma) in PBS at 37°C for 30 min. Wells were washed once in PBS before use. Cells (1.6×10^4^) labeled with 3.0 mM 2′7′-bis-(2-carboxyethyl)-5-(and-6)-carboxyfluorescein fluorescent dye (Invitrogen, Carlsbad, CA) were seeded into each ligand-coated well and incubated in a cell culture incubator for 30 min. The activating mAb KIM185 and function-blocking mAb LPM19c were also included in the assays (10 µg/ml each). Fluorescence measurements before and after washing steps were performed on a FL600 fluorescent plate reader (Bio-Tek Instruments, Winooski, VT). The % cell adhesion was calculated based on (fluorescence of bound cells after wash/total cell fluorescence before wash)×100.

### siRNA-silencing of kindlin-3 expression in K562 cells stably expressing integrin αMβ2

K562 cells stably expressing wild-type integrin αMβ2 [51] (referred herein as KM cells) were kindly provided by Dr. L. Zhang (University of Maryland, Baltimore, MD) and cultured in RPMI1640 medium containing 10% (v/v) heat-inactivated FCS and 100 IU/ml of penicillin and 100 µg/ml of streptomycin. Silencing of kindlin-3 expression in these cells was performed using a 3^rd^ generation lentiviral-based siRNA transduction system with GFP as the reporter (Applied Biological Materials, BC, Canada) [42]. The kindlin-3 siRNA sequence used was 5′-CCGAATTGTACACGAGTAT-3′. Stable cells expressing kindlin-3 (k3-KM) or control siRNA (ctrl-KM) were selected with 1.5 µg/mL puromycin. GFP expression in these cells was determined by flow cytometry. Knockdown efficiency and relative mRNA expression of kindlin-3 were determined by real-time PCR using the Power SYBR® Green Cells-to-CT™ kit (Ambion, Life technologies, Carlsbad, CA) on a CFX96™ real-time PCR detection system (Biorad laboratories, Hercules, CA). Kindlin-3 primer sequences are: (F) 5′-TTCCAGGCTGTGGCTGCCAT-3′ and 5′-CCCAGCCAAGACAACCTTGC-3′. Actin primer sequences are: (F) 5′-GACATGGAGAAAATCTGGCA-3′ and (R) 5′-TGGGGTGTTGAAGGTCTCAA-3′.

### Transfection of K562 cells with integrin αMβ2N329S

K562 cells stably transduced with kindlin-3-targeting or control siRNA were previously described [42]. These cells were cultured in RPMI1640 medium containing 10% (v/v) heat-inactivated FCS and 100 IU/ml of penicillin and 100 µg/ml of streptomycin. Cells (1×10^6^) were transfected with αM (8 µg) [48] and β2N329S (8 µg) [52] expression plasmids by electroporation (pulse voltage 1300, pulse width 10, pulse number 3) using a pipette-type microporator MP-100 (NanoEnTek Inc, Seoul, Korea) [53].

### Shear flow experiments

Shear flow experiments were performed using μ-Slide I^0.4^ Luer flow chamber (Ibidi GmbH, Germany). The channel of the flow chamber was coated with 7.5 µg/ml iC3b (Complement Technology) in PBS at 4°C overnight. Non-specific binding sites were blocked with 0.2% (w/v) PVP in PBS at RT for 1 h. The flow chamber was mounted on an inverted light microscope stage (Olympus, Center Valley, PA) in a custom-built plastic box connected to a temperature-controlled 37°C heater. The channel of the flow chamber was washed once in Buffer A (HBSS containing 1 mM Ca^2+^, 1 mM Mg^2+^, 5% (v/v) FBS, 10 mM HEPES, pH 7.4). Cells (6×10^5^) were re-suspended in 1 mL of Buffer A with or without activating mAb KIM185 (10 µg/mL) before infusion into the flow chamber at different flow rates using an automated syringe pump (Harvard Apparatus, Holliston, MA). At the end of the infusion, the number of adherent cells in four different fields (1 mm vs 1 mm, under 10×objective lens) along the center of the channel was counted. The average number of adherent cells per field is plotted against shear stress (dynes/cm^2^).

### Flow cytometry analyses

Flow cytometry analyses were performed as previously described [42]. In brief, cells expressing integrin αMβ2 or αMβ2N329S were stained with mAb LPM19c (10 µg/ml) in PBS at 4°C for 30 min. Cells were washed in PBS and incubated in PBS containing APC-conjugated goat anti-mouse IgG (1∶400) on ice for 30 min. Sample acquisitions were performed on a FACSCalibur flow cytometer (BD Bioscience) and data were analyzed using the Flowjo software (Tree Star Inc, Ashland, OR). Expression index (EI) was calculated by % cells gated positive (GP) X geo-mean fluorescence intensity (GM). For flow cytometry analyses of activated αMβ2 on ctrl-KM and k3-KM cells, cells were stained with mAb KIM127 (10 µg/ml) in the absence or presence of MnCl_2_ (1 mM) in complete RPMI1640 medium at 37°C for 30 min. Thereafter, cells were washed and incubated in complete RPMI1640 medium containing APC-conjugated goat anti-mouse IgG (1∶400) on ice for 30 min.

### Electric cell-substrate impedance sensing (ECIS) measurements

Each well of a 16-well E-plate® device (Acea Biosciences, San Diego, CA) with gold-electrodes was treated with dithiobis succinimidyl propionate (Pierce, Rockford, IL) (4 mg/mL) in DMSO for 30 min at RT [42]. Wells were washed twice in de-ionized H_2_O followed by coating with iC3b (7.5 µg/ml) or BSA (100 µg/ml) in PBS for 1 h at RT. K562 transfectants (8×10^4^ cells) were seeded into each well and AC impedance measurements (cell index) taken at 1 min intervals on a Real Time Cell Electronic System™ (Acea Biosciences) setup in a humidified CO_2_ cell culture incubator.

### Biochemical assays to measure the activities of Cdc42, Rac, and RhoA

Ctrl-KM or k3-KM cells were cultured in non-TC petri dishes in the absence of serum for 18 h. Serum-free condition was used to avoid potential contribution from growth factor-mediated intracellular signaling. From each group 10^7^ cells in serum-free medium were transferred into iC3b-coated TC dish and incubated for 15 min under culture conditions in the presence of mAb KIM185 (10 µg/ml). Cells were collected, lysed in 500 µl lysis buffer (1% v/v NP-40, 150 mM NaCl, 0.5 mM MgCl_2_, 0.15 mM CaCl_2_, 10 mM Tris, pH 8.0) containing protease and phosphatase inhibitors and incubated on ice for 20 min. Cell lysate was centrifuged to remove debris and nuclei. GST-Rhotekin-RBD or GST-PAK-PBD bead suspension (Cytoskeleton, Inc, Denver, CO) (20 µl each) was added to 200 µl of cell lysate and incubated for 3 h at 4°C with rotation. Beads were recovered by centrifugation followed by three washes in lysis buffer. Beads were boiled in SDS-sample buffer containing 40 mM DTT for 10 min to elute bound proteins.

### Immunoblotting

Proteins were resolved by SDS-PAGE under reducing conditions and electro-transferred onto Immobilon P membrane (Millipore, Bedford, MA). Immunoblotting was performed using relevant primary antibody and HRP-conjugated secondary antibody. Protein bands were detected by enhanced chemiluminescence using the ECLplus kit (GE Healthcare).

### Detection of phosphorylated Syk and Vav1

KM, ctrl-KM or k3-KM cells (5×10^6^) were placed in iC3b-coated TC dish in the presence of either 10 µg/ml of control IgG or mAb KIM185 at 37°C in a humidified CO_2_ cell culture incubator for 30 min. Cells were collected and lysed in lysis buffer (1% (v/v) Nonidet P-40, 150 mM NaCl and 10 mM Tris, pH 8.0) containing appropriate protease and phosphatase inhibitors. Immunoprecipitation was performed with either mouse anti-Syk or rabbit anti-Vav1 antibody with appropriate irrelevant IgG as control and protein A-Sepharose beads (GE Healthcare). Proteins were resolved by SDS-PAGE under reducing conditions.

To detect Tyr-phosphorylated Syk and Vav1, rabbit anti-Syk pY525/526 and mouse anti-phosphotyrosine (PY20) were respectively used in immunoblottings. To detect total immunoprecipitated Syk and Vav1 proteins, membranes were stripped of these antibodies in buffer containing 0.7% (v/v), β-mercaptoethanol, 2% (w/v) SDS and 62.5 mM Tris (pH 6.8) at 55°C for 30 min. The membranes were extensively washed followed by re-blotting with mouse anti-Syk or rabbit anti-Vav1 antibodies.

## Results

### Kindlin-3 is required for integrin αMβ2-mediated cell adhesion

To examine kindlin-3 function in integrin αMβ2-mediated adhesion, we made use of K562 cells that stably expressed integrin αMβ2 [17], [51]. These cells will henceforth be referred to as KM cells. KM cells were transduced with lentiviral-based control siRNA or kindlin-3 targeting siRNA and they will be referred to as ctrl-KM and k3-KM cells, respectively.

**Figure 1:**
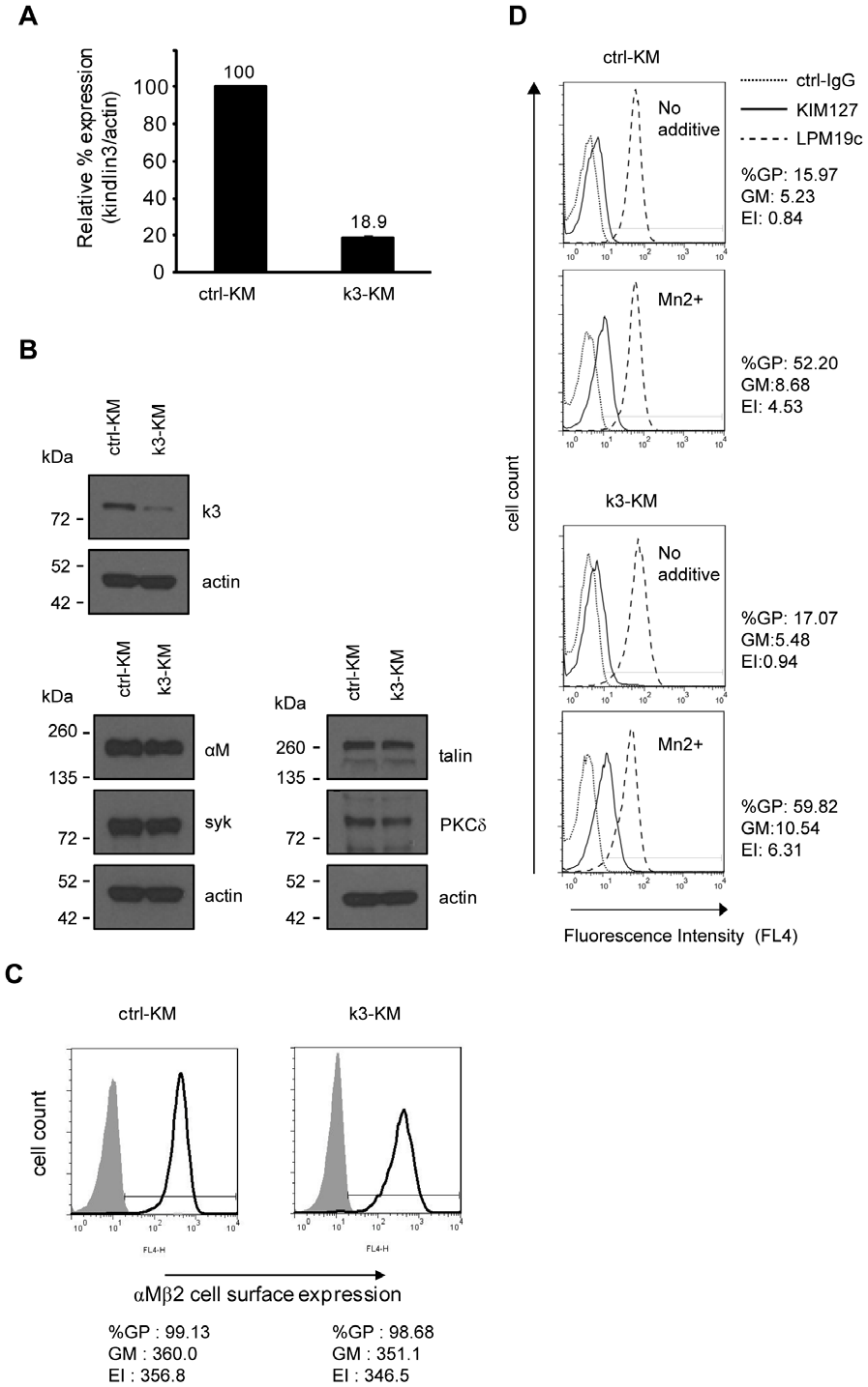
Knockdown of kindlin-3 expression in K562 cells expressing integrin αMβ2. (A) qPCR analyses of kindlin-3 mRNA expression level in ctrl-KM and k3-KM cells. (B) Expression levels of kindlin-3 and other proteins in these cells were determined by immunoblotting. Actin was used as loading control. (C) Cell surface expression of integrin αMβ2 was determined by flow cytometry. Shaded and open histograms represent control IgG and mAb LPM19c stainings, respectively. GP: gated positive; GM: geo-mean; EI: expression index. (D) To determine extracellular activation of integrin αMβ2 on ctrl-KM and k3-KM cells. Cells were treated with Mn^2+^ (1 mM) or without and stained with mAb KIM127 at 37°C. Control IgG (ctrl-IgG) and mAb LPM19c were included for each condition. The %GP, GM and EI of mAb KIM127 staining are shown. One representative experiment out of two independent experiments is shown.

Reduced expression of kindlin3 transcript and protein in k3-KM cells was verified by reverse transcription qPCR and western blot analyses, respectively (Fig. 1A & 1B). Expression levels of cytoplasmic proteins talin, Syk and PKCδ which have been reported to be important in integrin αMβ2 ligand-binding and signaling [17], [54], [55] were similar in ctrl-KM and k3-KM cells. Comparable levels of cell-surface expressed integrin αMβ2 in ctrl-KM and k3-KM cells were confirmed by flow cytometry analyses (Fig. 1C). In the presence of exogenous Mn^2+^, ctrl-KM and k3-KM showed comparable levels of staining for the mAb KIM127 that reports extended and activated β2 integrins (Fig. 1D) [49], [50]. Hence reduced kindlin-3 expression in k3-KM cells did not affect the capacity of integrin αMβ2 on these cells to undergo extracellular activation.

**Figure 2:**
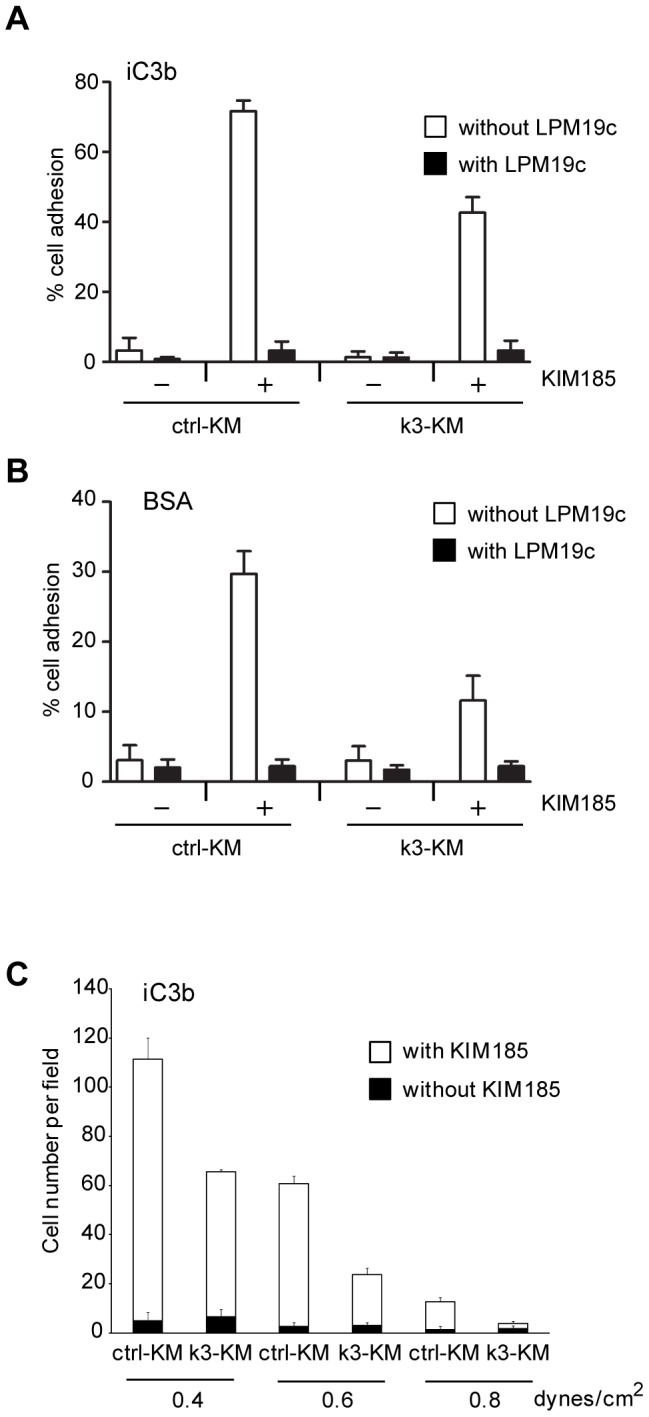
Reduced kindlin-3 expression diminished integrin αMβ2-mediated cell adhesion. (A) and (B) show adhesion data of ctrl-KM and k3-KM cells on iC3b and BSA, respectively. Each data point represents the mean ± SD of three independent experiments. mAbs LPM19c and KIM185 were used at 10 µg/ml each. (C) Shear flow analyses of ctrl-KM and k3-KM cells in flow chambers coated with iC3b. Each data point is the mean ± SD of number of cells in four fields and a representative plot of two independent experiments is shown.

The adhesive properties of ctrl-KM and k3-KM cells were assessed by performing static adhesion assay on immobilized integrin αMβ2 ligand iC3b (Fig. 2A). Although all cells bound to iC3b in the presence of β2-integrin activating mAb KIM185 [56], adhesion of k3-KM cells was reduced compared with ctrl-KM cells. Adhesion specificity mediated by integrin αMβ2 was demonstrated by including the function-blocking mAb LPM19c. Integrin αMβ2 is a promiscuous receptor with many ligands, including denatured proteins such as denatured BSA [7], [57]–[59]. The adhesion profiles of ctrl-KM and k3-KM cells on BSA were similar to that on iC3b (Fig. 2B). Kindlin-3 promotes adhesion strengthening that confers resistance of T cells to detachment forces [47]. We performed shear flow experiments using iC3b-coated μ-slide flow chambers (Fig. 2C). Under activating condition (with mAb KIM185), the number of ctrl-KM cells adhering to iC3b at 0.4 and 0.6 dynes/cm^2^ was significantly higher than that of k3-KM cells. All cells adhered poorly to iC3b in the absence of activating condition.

Collectively, these data suggest that kindlin-3 is required for integrin αMβ2-mediated firm adhesion of cells. The reduced avidity of k3-KM cells on integrin αMβ2 ligands is unlikely due to a lack of integrin αMβ2 affinity up-regulation because the activating mAb KIM185 was used to bypass the need for inside-out β2-integrin activation.

### A role for kindlin-3 in integrin αMβ2 outside-in signaling that regulates cell spreading

**Figure 3:**
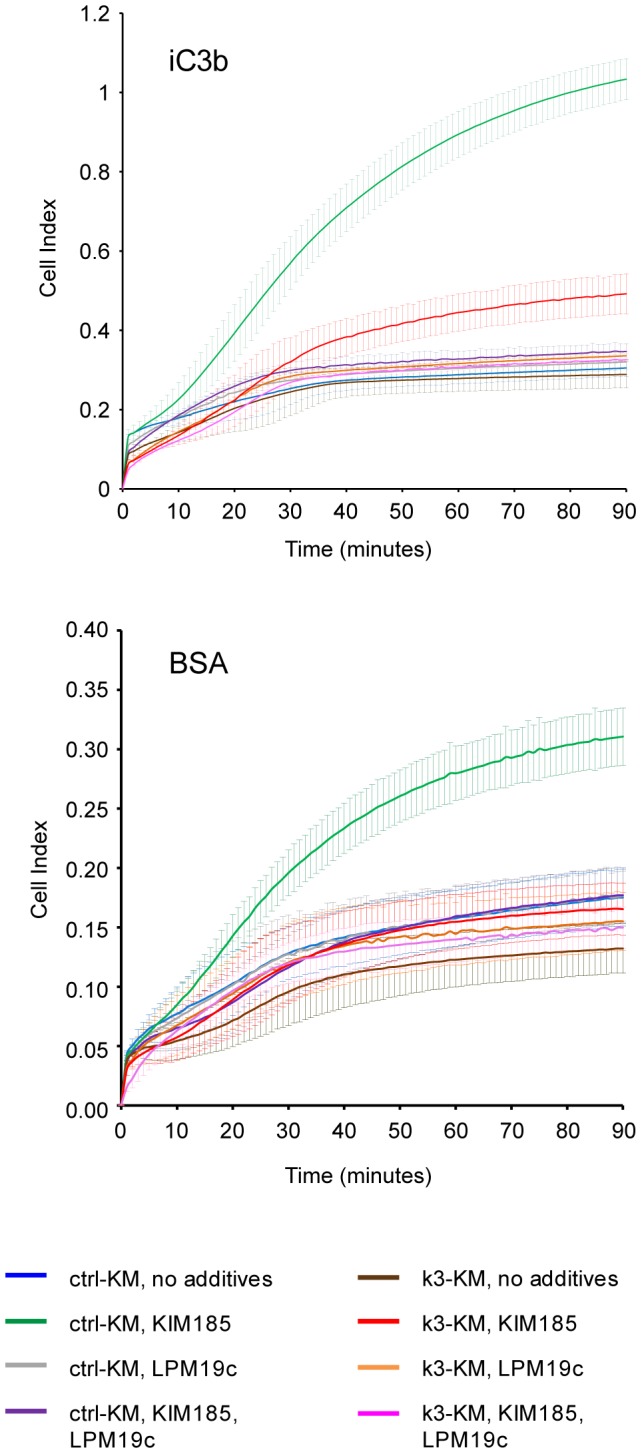
Kindlin-3 is required for integrin αMβ2-mediated cell spreading. ECIS measurements of ctrl-KM and k3-KM cells spreading on iC3b or BSA. Each data point represents the mean ± SD of technical triplicates at 1 min intervals. mAbs LPM19c and KIM185 were used at 10 µg/ml each. A plot of a representative experiment from three independent experiments is shown for each ligand.

We have shown that kindlin-3 plays a role in outside-in signaling of integrins αLβ2 and αIIbβ3 [42]. Using the real-time electrical cell-substrate impedance sensing (ECIS) method [42], we analyzed cell spreading of ctrl-KM and k3-KM cells on iC3b and BSA (Fig. 3). Under activating condition (with mAb KIM185), ctrl-KM cells but not k3-KM cells adhered and spread effectively on either iC3b or BSA over a period of 90 min. Addition of function-blocking mAb LPM19c abrogated cell adhesion and spreading to levels comparable to that of the non-activating condition.

**Figure 4:**
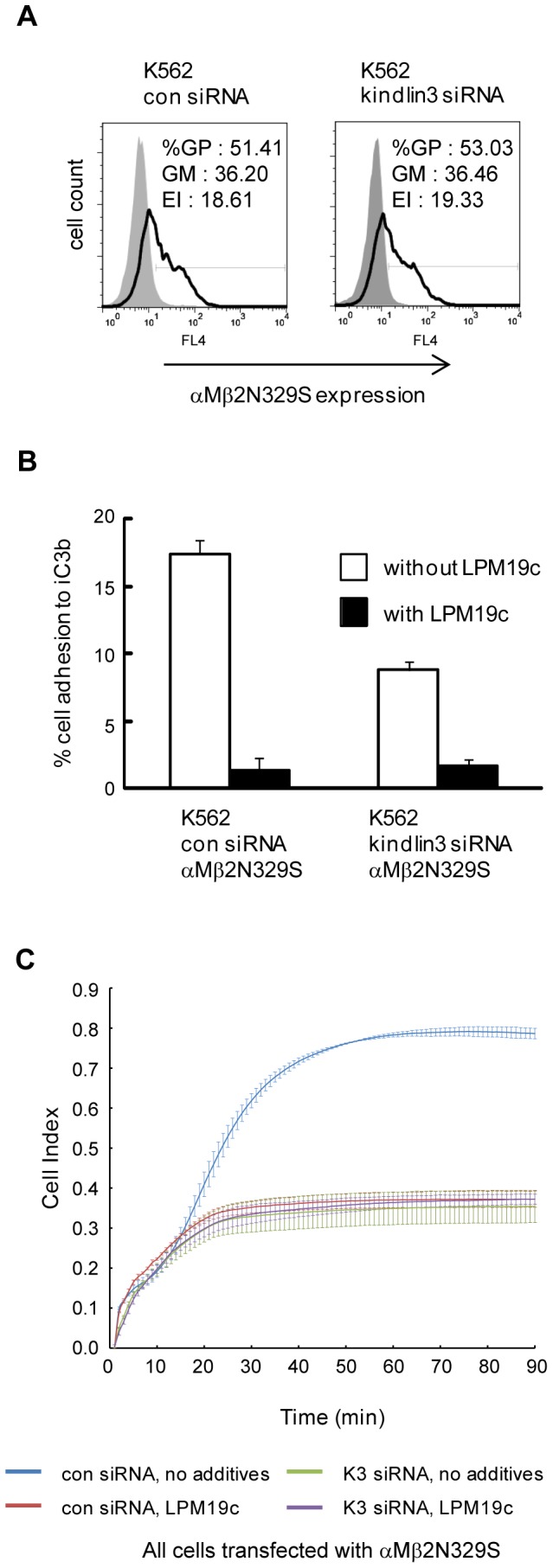
Kindlin-3 is involved in integrin αMβ2 outside-in signaling. (A) Flow cytometry analyses of αMβ2N329S in cells transduced with control or kindlin-3-targeting siRNA. Shaded and open histograms represent control IgG and LPM19c stainings, respectively. GP: gated positive; GM: geo-mean; EI: expression index. (B) Cell adhesion assay on iC3b. (C) ECIS measurements on iC3b. In (B) and (C), each data point represents mean ± SD of technical triplicates. mAb LPM19c was used at 10 µg/ml. (A–C), a single representative experiment from three independent experiments is shown.

We further verified a role of kindlin-3 in integrin αMβ2 outside-in signaling by using a constitutively activated integrin mutant αMβ2N329S. The mutation N329S in the integrin β2 subunit induces high ligand-binding affinity in integrin αLβ2 [52]. K562 cells stably expressing either control siRNA or kindlin-3 targeting siRNA [42] were transfected with integrin αMβ2N329S. The expression level of αMβ2N329S on transfectant was determined by flow cytometry analysis (Fig. 4A). Static adhesion assays showed that whereas both αMβ2N329S-expressing control siRNA and kindlin3-targeting siRNA cells adhered constitutively to iC3b, the level of cell adhesion was lower for the latter (Fig. 4B). Adhesion specificity mediated by αMβ2N329S was demonstrated using mAb LPM19c. ECIS experiments also showed defective cell adhesion and spreading on iC3b of kindlin3-targeting siRNA cells despite expressing a constitutively activated αMβ2N329S (Fig. 4C). Taken together these data support a role of kindlin-3 in integrin αMβ2-mediated outside-in signaling and cell spreading.

### Kindlin-3 in integrin αMβ2 outside-in signaling that activates Rho GTPases

**Figure 5:**
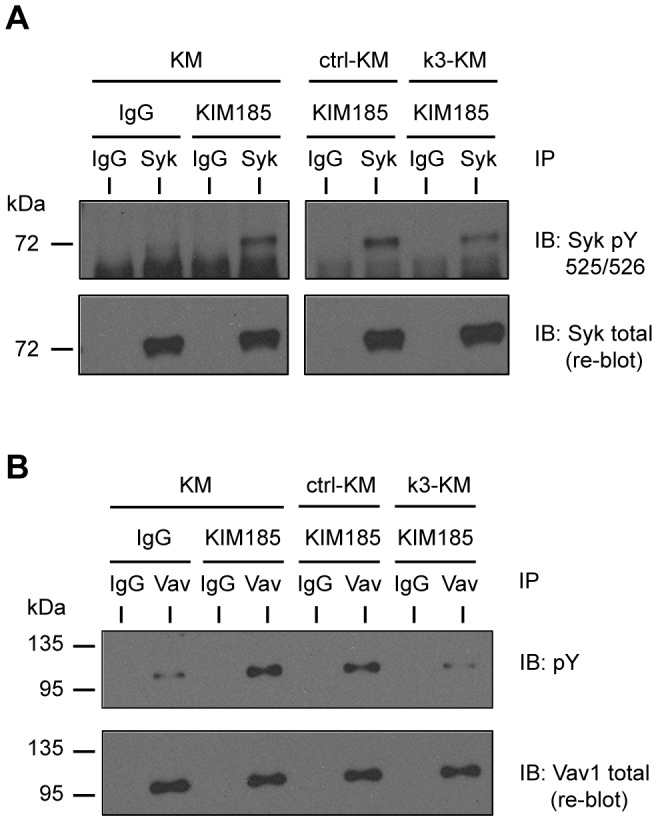
Kindlin-3 regulates the integrin αMβ2-Syk-Vav1 signaling axis. KM, ctrl-KM and k3-KM cells were seeded into iC3b-coated TC dishes in the presence of irrelevant mouse IgG (IgG) or mAb KIM185 (10 µg/ml each) and incubated under culture conditions for 30 min. Cells were harvested and lysed followed by immunoprecipitation (IP) using either anti-Syk or anti-Vav1 antibody with mouse IgG or rabbit anti-GST antibody as irrelevant IgG, respectively. Tyr-phosphorylated Syk and Vav1 were probed as described under materials and methods. IB: immunoblotting. A representative experiment from two independent experiments is shown.

Studies have shown that kindlin-3 is required for integrin-mediated cell spreading [27], [30], [42]. In hematopoietic cells, the non-receptor tyrosine kinase Syk plays an important role in early signaling events derived from β2 integrins and it is important for spreading in polymorphonuclear leukocytes [20], [55]. We tested integrin αMβ2-mediated phosphorylation of Syk in KM, ctrl-KM and k3-KM cells (Fig. 5A). All cells were seeded onto iC3b but were treated with either control IgG or mAb KIM185. Cells were incubated under culture conditions for 30 min before harvesting for immunoprecipitation assays. Activated Syk in immunoprecipitates was detected using anti-phospho Tyr525/526 Syk. Activated Syk was detected in KM cells that were plated on iC3b in the presence of mAb KIM185, but not control IgG. Under the same conditions, Syk activation was also observed in ctrl-KM cells, but the level of activation was reduced in k3-KM cells.

Syk associates with and phosphorylates Vav guanine exchange factors [60], [61]. The Syk-Vav signaling axis has been shown to be important for β2 integrin-mediated neutrophil adhesion and migration [62], [63]. Thus we examined the phosphorylation status of Vav1, which is predominantly expressed in hematopoietic cells [64]. Basal tyrosine phosphorylation of Vav1 was detected in KM cells plated on iC3b, and the level of phosphorylation was enhanced when mAb KIM185 was included (Fig. 5B). Vav1 phosphorylation was also detected in ctrl-KM cells plated on iC3b in the presence of mAb KIM185. By contrast, Vav1 phosphorylation was at a basal level in k3-KM cells under the same conditions.

**Figure 6:**
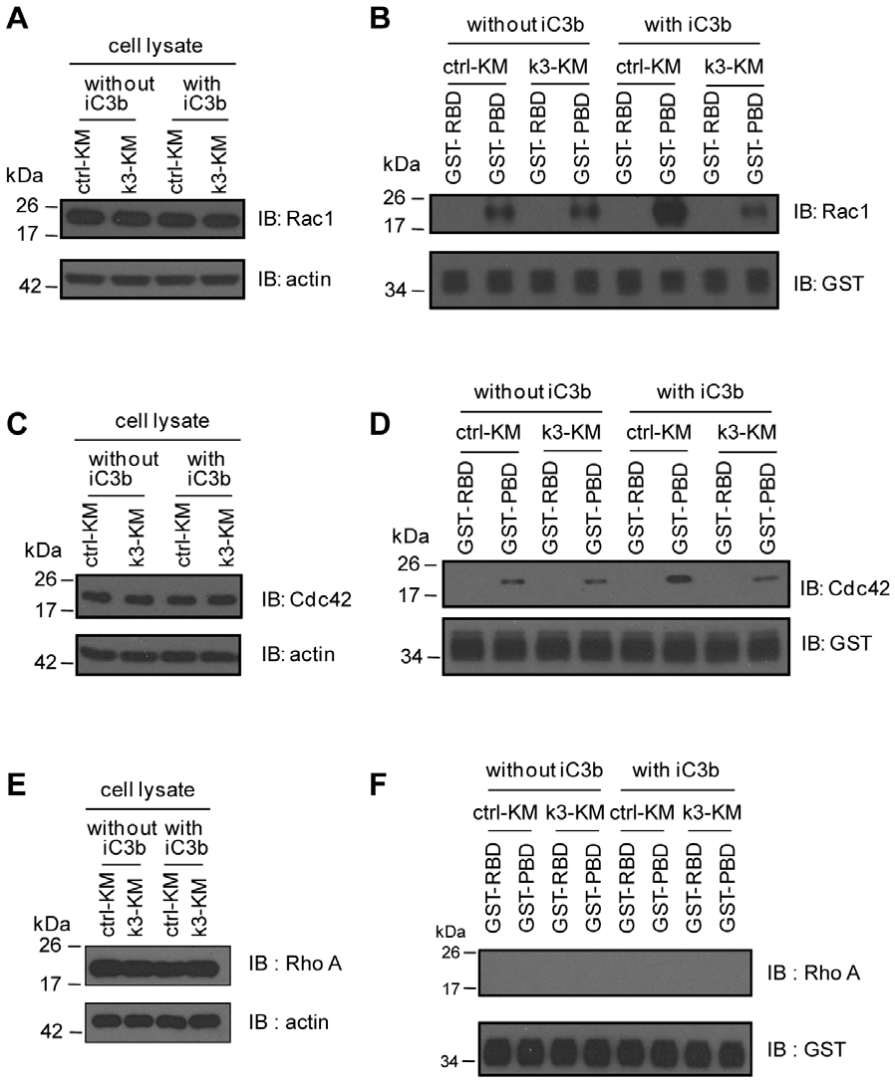
Integrin αMβ2-induced RhoGTPase activation involves kindlin-3. Ctrl-KM and k3-KM cells were allowed to adhere to iC3b-coated TC dishes in the presence of mAb KIM185 (10 µg/ml). (A), (C) and (E) are immunoblots of cell lysates for Rac1, Cdc42 and RhoA, respectively. (B), (D) and (F) are pull-down experiments using cell lysates and RBD or PBD-conjugated beads. IB: immunoblotting. A representative experiment from two independent experiments is shown.

Vav proteins are known to regulate Rho GTPases, which in turn control the actin dynamics [64]. We therefore examined the activation of Rac1, Cdc42 and RhoA in ctrl-KM and k3-KM cells by GST-RBD and GST-PBD pull-down assays. The mAb KIM185 was included in all conditions and there was no significant difference between ctrl-KM and k3-KM cells in terms of Rac1, Cdc42 or RhoA expression (Fig. 6A, C & E). Comparable basal levels of activated Rac1 or Cdc42 were detected in ctrl-KM and k3-KM cells in the absence of iC3b (Fig. 6B & D). In the presence of iC3b, higher levels of activated Rac1 and Cdc42 were detected in ctrl-KM cells compared with k3-KM cells. We failed to detect activated RhoA in these cells under all conditions (Fig. 6F). Collectively, these data suggest that kindlin-3 plays an important role in integrin αMβ2 outside-in signaling that regulates cytoskeletal remodeling.

## Discussion

Kindlin-3 functions as a co-activator of β2 integrins and it induces a high-affinity integrin αLβ2 [30], [65]. We have previously shown a role for kindlin-3 in integrin αLβ2 outside-in signaling [42]. In this study, we provide evidence that kindlin-3 is involved in the integrin αMβ2-Syk-Vav1 signaling axis that regulates Rho GTPases Rac1 and Cdc42. We observed defective spreading on iC3b of KM cells with reduced kindlin-3 expression. Activation of Syk and Vav1 in these cells was marginal and downstream activities of Rac1 and Cdc42 were reduced. Although cell adhesion and spreading is a complex process involving integrin avidity regulation, it is unlikely that a lack of activated integrin αMβ2 in these cells accounts for the observed deficiencies. We used either activating mAb KIM185 or expressed constitutively activated integrin αMβ2 mutant (αMβ2N329S) in these studies. Because both methods bypass inside-out signaling, defective integrin αMβ2 activation as a result of reduced kindlin-3 expression is unlikely.

It is evident that KM cells with reduced kindlin-3 expression showed impaired outside-in signaling. How does kindlin-3 regulate integrin αMβ2-Syk signaling? Clustering of β2 integrins is known to induce Syk activation [66], and co-localization of Syk with the β2 integrins at the lamellipodium of neutrophils during the early stages of spreading has been reported [67]. We have also shown that kindlin-3 promotes integrin αLβ2 micro-clustering [42]. Hence, kindlin-3 could stabilize or enhance integrin αMβ2 micro-clustering when KM cells were plated on immobilized iC3b. Studies on kindlin-2 have shown that its PH domain binds phosphatidyl-inositol-4,5-bisphosphate (PIP2) and PIP3, albeit with different affinities [35], [36]. Total internal reflection fluorescence (TIRF) imaging of EGFP-kindlin-3 transfected LADIII lymphocytes that were plated on fibrinogen showed the recruitment of EGFP-kindlin-3 at contact sites with the substrate [29]. Taken together, the recruitment of kindlin-3 to PIP2/PIP3-enriched integrin-ligand contact areas of the plasma membrane could potentially lead to clustering of integrin αMβ2 followed by the activation of Syk.

Integrins regulate the activities of Rho GTPases [68]. Rac and Cdc42 induce the formation of lamellipodia and filopodia, respectively [69]–[71]. Rho regulates the formation of stress fibers, the assembly of focal adhesions, and cell contractility [72]. We have shown the importance of kindlin-3 in integrin αMβ2-induced activation of Rac1 and Cdc42 in KM cells. However, we were unable to detect RhoA activation. Up-regulation of Rho activity has been reported in integrin αVβ3 expressing K562 cells plated on vitronectin, but the phorbol ester PMA was used as the activating agent [73]. Different integrins also regulate the activities of distinct Rho GTPases. For example, over-expressed β3 and β1 integrins in CHO cells enhanced Rho and Rac activities, respectively [74]. Importantly, Rac1 suppresses the activity of RhoA and vice versa [75], [76]. It is conceivable that the activation of Rac and Cdc42 with concomitant inhibition of RhoA is important during the early stages of cell spreading when membrane protrusions are essential whereas the reverse occurs in fully spread cells to form stress fibers and focal adhesions [77].

Taken together, our data show that kindlin-3 is required for integrin αMβ2-mediated outside-in signaling that leads to the activation of Rac1 and Cdc42. Whether the involvement of kindlin-3 in the Syk-Vav1-Rac1/Cdc42 signaling axis is a general outside-in signaling mechanism for all β2 integrins remains to be determined. Our data also suggest that kindlin-3 plays an important role in the early phase of integrin αMβ2-mediated cell spreading which corroborates well with the observations that kindlin-3 is localized to lamellipodia rather than mature focal adhesion sites of HUVEC spreading on fibronectin [27] (and unpublished data from our group). Previously, we reported the interaction between kindlin-3 and the receptor for activated-C kinase (RACK1) [42]. RACK1 is a scaffold protein that has been shown to localize to nascent focal adhesion sites [78]–[80]. Kindlin-3 and RACK1 interaction was also detected in KM cells (data not shown). Future work will examine the interplay between these molecules in integrin-induced cytoskeletal remodeling.

## References

[1] Hynes RO (2002) Integrins: bidirectional, allosteric signaling machines. Cell 110: 673–687. 12297042 10.1016/s0092-8674(02)00971-6

[2] Arnaout MA, Goodman SL, Xiong JP (2007) Structure and mechanics of integrin-based cell adhesion. Curr Opin Cell Biol 19: 495–507. 17928215 10.1016/j.ceb.2007.08.002 PMC2443699

[3] Luo BH, Carman CV, Springer TA (2007) Structural basis of integrin regulation and signaling. Annu Rev Immunol 25: 619–647. 17201681 10.1146/annurev.immunol.25.022106.141618 PMC1952532

[4] Campbell ID, Humphries MJ (2011) Integrin structure, activation, and interactions. Cold Spring Harb Perspect Biol 3. 10.1101/cshperspect.a004994 PMC3039929 21421922

[5] Puklin-Faucher E, Sheetz MP (2009) The mechanical integrin cycle. J Cell Sci 122: 179–186. 19118210 10.1242/jcs.042127 PMC6518156

[6] McEver RP, Zhu C (2007) A catch to integrin activation. Nat Immunol 8: 1035–1037. 17878912 10.1038/ni1007-1035

[7] Tan SM (2012) The leucocyte β2 (CD18) integrins: the structure, functional regulation and signalling properties. Bioscience Reports 32: 241–269. 22458844 10.1042/BSR20110101

[8] Larson RS, Springer TA (1990) Structure and function of leukocyte integrins. Immunol Rev 114: 181–217. 2196220 10.1111/j.1600-065x.1990.tb00565.x

[9] Graff JC, Jutila MA (2007) Differential regulation of CD11b on γδ T cells and monocytes in response to unripe apple polyphenols. J Leukoc Biol 82: 603–607. 17540733 10.1189/jlb.0207125

[10] Springer T, Galfre G, Secher DS, Milstein C (1979) Mac-1: a macrophage differentiation antigen identified by monoclonal antibody. Eur J Immunol 9: 301–306. 89034 10.1002/eji.1830090410

[11] Beller DI, Springer TA, Schreiber RD (1982) Anti-Mac-1 selectively inhibits the mouse and human type three complement receptor. J Exp Med 156: 1000–1009. 7153706 10.1084/jem.156.4.1000 PMC2186827

[12] Arnaout MA, Todd RF, Dana N, Melamed J, Schlossman SF, et al (1983) Inhibition of phagocytosis of complement C3- or immunoglobulin G-coated particles and of C3bi binding by monoclonal antibodies to a monocyte-granulocyte membrane glycoprotein (Mol). J Clin Invest 72: 171–179. 6874946 10.1172/JCI110955 PMC1129172

[13] Ding ZM, Babensee JE, Simon SI, Lu H, Perrard JL, et al (1999) Relative contribution of LFA-1 and Mac-1 to neutrophil adhesion and migration. J Immunol 163: 5029–5038. 10528208

[14] Chavakis T, Bierhaus A, Al-Fakhri N, Schneider D, Witte S, et al (2003) The pattern recognition receptor (RAGE) is a counterreceptor for leukocyte integrins: a novel pathway for inflammatory cell recruitment. J Exp Med 198: 1507–1515. 14623906 10.1084/jem.20030800 PMC2194124

[15] Shi C, Zhang X, Chen Z, Sulaiman K, Feinberg MW, et al (2004) Integrin engagement regulates monocyte differentiation through the forkhead transcription factor Foxp1. J Clin Invest 114: 408–418. 15286807 10.1172/JCI21100 PMC484980

[16] Shi C, Sakuma M, Mooroka T, Liscoe A, Gao H, et al (2008) Down-regulation of the forkhead transcription factor Foxp1 is required for monocyte differentiation and macrophage function. Blood 112: 4699–4711. 18799727 10.1182/blood-2008-01-137018 PMC2597137

[17] Xue ZH, Zhao CQ, Chua GL, Tan SW, Tang XY, et al (2010) Integrin αMβ2 clustering triggers phosphorylation and activation of protein kinase Cδ that regulates transcription factor Foxp1 expression in monocytes. J Immunol 184: 3697–3709. 20190138 10.4049/jimmunol.0903316

[18] Mayadas TN, Cullere X (2005) Neutrophil β2 integrins: moderators of life or death decisions. Trends Immunol 26: 388–395. 15922663 10.1016/j.it.2005.05.002

[19] Skoberne M, Somersan S, Almodovar W, Truong T, Petrova K, et al (2006) The apoptotic-cell receptor CR3, but not αVβ5, is a regulator of human dendritic-cell immunostimulatory function. Blood 108: 947–955. 16614246 10.1182/blood-2005-12-4812 PMC1895855

[20] Han C, Jin J, Xu S, Liu H, Li N, et al (2010) Integrin CD11b negatively regulates TLR-triggered inflammatory responses by activating Syk and promoting degradation of MyD88 and TRIF via Cbl-b. Nat Immunol 11: 734–742. 20639876 10.1038/ni.1908

[21] Tadokoro S, Shattil SJ, Eto K, Tai V, Liddington RC, et al (2003) Talin binding to integrin β tails: a final common step in integrin activation. Science 302: 103–106. 14526080 10.1126/science.1086652

[22] Critchley DR, Gingras AR (2008) Talin at a glance. J Cell Sci 121: 1345–1347. 18434644 10.1242/jcs.018085

[23] Shattil SJ, Kim C, Ginsberg MH (2010) The final steps of integrin activation: the end game. Nat Rev Mol Cell Biol 11: 288–300. 20308986 10.1038/nrm2871 PMC3929966

[24] Meves A, Stremmel C, Gottschalk K, Fassler R (2009) The Kindlin protein family: new members to the club of focal adhesion proteins. Trends Cell Biol 19: 504–513. 19766491 10.1016/j.tcb.2009.07.006

[25] Malinin NL, Plow EF, Byzova TV (2010) Kindlins in FERM adhesion. Blood 115: 4011–4017. 20228270 10.1182/blood-2009-10-239269 PMC2875095

[26] Moser M, Nieswandt B, Ussar S, Pozgajova M, Fassler R (2008) Kindlin-3 is essential for integrin activation and platelet aggregation. Nat Med 14: 325–330. 18278053 10.1038/nm1722

[27] Bialkowska K, Ma YQ, Bledzka K, Sossey-Alaoui K, Izem L, et al (2010) The integrin co-activator kindlin-3 is expressed and functional in a non-hematopoietic cell, the endothelial cell. J Biol Chem 285: 18640–18649. 20378539 10.1074/jbc.M109.085746 PMC2881789

[28] Mory A, Feigelson SW, Yarali N, Kilic SS, Bayhan GI, et al (2008) Kindlin-3: a new gene involved in the pathogenesis of LAD-III. Blood 112: 2591. 18779414 10.1182/blood-2008-06-163162

[29] Malinin NL, Zhang L, Choi J, Ciocea A, Razorenova O, et al (2009) A point mutation in KINDLIN3 ablates activation of three integrin subfamilies in humans. Nat Med 15: 313–318. 19234460 10.1038/nm.1917 PMC2857384

[30] Moser M, Bauer M, Schmid S, Ruppert R, Schmidt S, et al (2009) Kindlin-3 is required for β2 integrin-mediated leukocyte adhesion to endothelial cells. Nat Med 15: 300–305. 19234461 10.1038/nm.1921

[31] Svensson L, Howarth K, McDowall A, Patzak I, Evans R, et al (2009) Leukocyte adhesion deficiency-III is caused by mutations in KINDLIN3 affecting integrin activation. Nat Med 15: 306–312. 19234463 10.1038/nm.1931 PMC2680140

[32] McDowall A, Svensson L, Stanley P, Patzak I, Chakravarty P, et al (2010) Two mutations in the KINDLIN3 gene of a new leukocyte adhesion deficiency III patient reveal distinct effects on leukocyte function in vitro. Blood 115: 4834–4842. 20357244 10.1182/blood-2009-08-238709

[33] Goult BT, Bouaouina M, Harburger DS, Bate N, Patel B, et al (2009) The structure of the N-terminus of kindlin-1: a domain important for αIIbβ3 integrin activation. J Mol Biol 394: 944–956. 19804783 10.1016/j.jmb.2009.09.061 PMC2963925

[34] Bouaouina M, Goult BT, Huet-Calderwood C, Bate N, Brahme NN, et al (2012) A conserved lipid-binding loop in the kindlin FERM F1 domain is required for kindlin-mediated αIIbβ3 integrin co-activation. J Biol Chem 287: 6979–6990. 22235127 10.1074/jbc.M111.330845 PMC3293583

[35] Qu H, Tu Y, Shi X, Larjava H, Saleem MA, et al (2011) Kindlin-2 regulates podocyte adhesion and fibronectin matrix deposition through interactions with phosphoinositides and integrins. J Cell Sci 124: 879–891. 21325030 10.1242/jcs.076976 PMC3048888

[36] Liu J, Fukuda K, Xu Z, Ma YQ, Hirbawi J, et al (2011) Structural basis of phosphoinositide binding to kindlin-2 protein pleckstrin homology domain in regulating integrin activation. J Biol Chem 286: 43334–43342. 22030399 10.1074/jbc.M111.295352 PMC3234820

[37] Perera HD, Ma YQ, Yang J, Hirbawi J, Plow EF, et al (2011) Membrane binding of the N-terminal ubiquitin-like domain of kindlin-2 is crucial for its regulation of integrin activation. Structure 19: 1664–1671. 22078565 10.1016/j.str.2011.08.012 PMC3217186

[38] Moser M, Legate KR, Zent R, Fassler R (2009) The tail of integrins, talin, and kindlins. Science 324: 895–899. 19443776 10.1126/science.1163865

[39] Harburger DS, Bouaouina M, Calderwood DA (2009) Kindlin-1 and -2 directly bind the C-terminal region of β integrin cytoplasmic tails and exert integrin-specific activation effects. J Biol Chem 284: 11485–11497. 19240021 10.1074/jbc.M809233200 PMC2670154

[40] Montanez E, Ussar S, Schifferer M, Bosl M, Zent R, et al (2008) Kindlin-2 controls bidirectional signaling of integrins. Genes Dev 22: 1325–1330. 18483218 10.1101/gad.469408 PMC2377186

[41] Tu Y, Wu S, Shi X, Chen K, Wu C (2003) Migfilin and Mig-2 link focal adhesions to filamin and the actin cytoskeleton and function in cell shape modulation. Cell 113: 37–47. 12679033 10.1016/s0092-8674(03)00163-6

[42] Feng C, Li YF, Yau YH, Lee HS, Tang XY, et al (2012) Kindlin-3 mediates integrin αLβ2 outside-in signaling, and it interacts with scaffold protein receptor for activated-C kinase 1 (RACK1). J Biol Chem 287: 10714–10726. 22334666 10.1074/jbc.M111.299594 PMC3322817

[43] Yu Y, Wu J, Wang Y, Zhao T, Ma B, et al (2012) Kindlin 2 forms a transcriptional complex with β-catenin and TCF4 to enhance Wnt signalling. EMBO reports 13: 750–758. 22699938 10.1038/embor.2012.88 PMC3410388

[44] Bouaouina M, Calderwood DA (2011) Kindlins. Current biology : CB 21: R99–101. 21300280 10.1016/j.cub.2010.12.002

[45] Has C, Herz C, Zimina E, Qu HY, He Y, et al (2009) Kindlin-1 is required for RhoGTPase-mediated lamellipodia formation in keratinocytes. Am J Pathol 175: 1442–1452. 19762715 10.2353/ajpath.2009.090203 PMC2751541

[46] Jung GY, Park YJ, Han JS (2011) Mediation of Rac1 activation by kindlin-2: an essential function in osteoblast adhesion, spreading, and proliferation. J Cell Biochem 112: 2541–2548. 21590706 10.1002/jcb.23178

[47] Manevich-Mendelson E, Feigelson SW, Pasvolsky R, Aker M, Grabovsky V, et al (2009) Loss of kindlin-3 in LAD-III eliminates LFA-1 but not VLA-4 adhesiveness developed under shear flow conditions. Blood 114: 2344–2353. 19617577 10.1182/blood-2009-04-218636

[48] Tang ML, Kong LS, Law SK, Tan SM (2006) Down-regulation of integrin αMβ2 ligand-binding function by the urokinase-type plasminogen activator receptor. Biochem Biophys Res Commun 348: 1184–1193. 16905120 10.1016/j.bbrc.2006.07.179

[49] Stephens P, Romer JT, Spitali M, Shock A, Ortlepp S, et al (1995) KIM127, an antibody that promotes adhesion, maps to a region of CD18 that includes cysteine-rich repeats. Cell Adhes Commun 3: 375–384. 8640375 10.3109/15419069509081292

[50] Beglova N, Blacklow SC, Takagi J, Springer TA (2002) Cysteine-rich module structure reveals a fulcrum for integrin rearrangement upon activation. Nat Struct Biol 9: 282–287. 11896403 10.1038/nsb779

[51] Xiong YM, Chen J, Zhang L (2003) Modulation of CD11b/CD18 adhesive activity by its extracellular, membrane-proximal regions. J Immunol 171: 1042–1050. 12847278 10.4049/jimmunol.171.2.1042

[52] Cheng M, Foo SY, Shi ML, Tang RH, Kong LS, et al (2007) Mutation of a conserved asparagine in the I-like domain promotes constitutively active integrins αLβ2 and αIIbβ3. J Biol Chem 282: 18225–18232. 17468108 10.1074/jbc.M701386200

[53] Tang XY, Li YF, Tan SM (2008) Intercellular adhesion molecule-3 binding of integrin αLβ2 requires both extension and opening of the integrin headpiece. J Immunol 180: 4793–4804. 18354203 10.4049/jimmunol.180.7.4793

[54] Lim J, Wiedemann A, Tzircotis G, Monkley SJ, Critchley DR, et al (2007) An essential role for talin during αMβ2-mediated phagocytosis. Mol Biol Cell 18: 976–985. 17202407 10.1091/mbc.E06-09-0813 PMC1805113

[55] Mocsai A, Abram CL, Jakus Z, Hu Y, Lanier LL, et al (2006) Integrin signaling in neutrophils and macrophages uses adaptors containing immunoreceptor tyrosine-based activation motifs. Nat Immunol 7: 1326–1333. 17086186 10.1038/ni1407 PMC4698344

[56] Andrew D, Shock A, Ball E, Ortlepp S, Bell J, et al (1993) KIM185, a monoclonal antibody to CD18 which induces a change in the conformation of CD18 and promotes both LFA-1- and CR3-dependent adhesion. Eur J Immunol 23: 2217–2222. 7690325 10.1002/eji.1830230925

[57] Davis GE (1992) The Mac-1 and p150,95 β2 integrins bind denatured proteins to mediate leukocyte cell-substrate adhesion. Exp Cell Res 200: 242–252. 1572393 10.1016/0014-4827(92)90170-d

[58] Tan SM, Hyland RH, Al-Shamkhani A, Douglass WA, Shaw JM, et al (2000) Effect of integrin β2 subunit truncations on LFA-1 (CD11a/CD18) and Mac-1 (CD11b/CD18) assembly, surface expression, and function. J Immunol 165: 2574–2581. 10946284 10.4049/jimmunol.165.5.2574

[59] Shaw JM, Al-Shamkhani A, Boxer LA, Buckley CD, Dodds AW, et al (2001) Characterization of four CD18 mutants in leucocyte adhesion deficient (LAD) patients with differential capacities to support expression and function of the CD11/CD18 integrins LFA-1, Mac-1 and p150,95. Clin Exp Immunol 126: 311–318. 11703376 10.1046/j.1365-2249.2001.01661.x PMC1906209

[60] Deckert M, Tartare-Deckert S, Couture C, Mustelin T, Altman A (1996) Functional and physical interactions of Syk family kinases with the Vav proto-oncogene product. Immunity 5: 591–604. 8986718 10.1016/s1074-7613(00)80273-3

[61] Miranti CK, Leng L, Maschberger P, Brugge JS, Shattil SJ (1998) Identification of a novel integrin signaling pathway involving the kinase Syk and the guanine nucleotide exchange factor Vav1. Current biology: CB 8: 1289–1299. 9843681 10.1016/s0960-9822(07)00559-3

[62] Schymeinsky J, Sindrilaru A, Frommhold D, Sperandio M, Gerstl R, et al (2006) The Vav binding site of the non-receptor tyrosine kinase Syk at Tyr 348 is critical for β2 integrin (CD11/CD18)-mediated neutrophil migration. Blood 108: 3919–3927. 16882714 10.1182/blood-2005-12-030387

[63] Gakidis MA, Cullere X, Olson T, Wilsbacher JL, Zhang B, et al (2004) Vav GEFs are required for β2 integrin-dependent functions of neutrophils. J Cell Biol 166: 273–282. 15249579 10.1083/jcb.200404166 PMC2172310

[64] Bustelo XR (2000) Regulatory and signaling properties of the Vav family. Mol Cell Biol 20: 1461–1477. 10669724 10.1128/mcb.20.5.1461-1477.2000 PMC85310

[65] Lefort CT, Rossaint J, Moser M, Petrich BG, Zarbock A, et al (2012) Distinct roles for talin-1 and kindlin-3 in LFA-1 extension and affinity regulation. Blood 119: 4275–4282. 22431571 10.1182/blood-2011-08-373118 PMC3359742

[66] Yan SR, Huang M, Berton G (1997) Signaling by adhesion in human neutrophils: activation of the p72syk tyrosine kinase and formation of protein complexes containing p72syk and Src family kinases in neutrophils spreading over fibrinogen. J Immunol 158: 1902–1910. 9029132

[67] Mocsai A, Zhou M, Meng F, Tybulewicz VL, Lowell CA (2002) Syk is required for integrin signaling in neutrophils. Immunity 16: 547–558. 11970878 10.1016/s1074-7613(02)00303-5

[68] Kjoller L, Hall A (1999) Signaling to Rho GTPases. Experimental cell research 253: 166–179. 10579921 10.1006/excr.1999.4674

[69] Jaffe AB, Hall A (2005) Rho GTPases: biochemistry and biology. Annual review of cell and developmental biology 21: 247–269. 10.1146/annurev.cellbio.21.020604.150721 16212495

[70] Gupton SL, Gertler FB (2007) Filopodia: the fingers that do the walking. Science's STKE: signal transduction knowledge environment 2007: re5. 10.1126/stke.4002007re5 17712139

[71] Ridley AJ, Paterson HF, Johnston CL, Diekmann D, Hall A (1992) The small GTP-binding protein rac regulates growth factor-induced membrane ruffling. Cell 70: 401–410. 1643658 10.1016/0092-8674(92)90164-8

[72] Ridley AJ, Hall A (1992) The small GTP-binding protein rho regulates the assembly of focal adhesions and actin stress fibers in response to growth factors. Cell 70: 389–399. 1643657 10.1016/0092-8674(92)90163-7

[73] Butler B, Williams MP, Blystone SD (2003) Ligand-dependent activation of integrin αvβ3. J Biol Chem 278: 5264–5270. 12446696 10.1074/jbc.M206997200

[74] Miao H, Li S, Hu YL, Yuan S, Zhao Y, et al (2002) Differential regulation of Rho GTPases by β1 and β3 integrins: the role of an extracellular domain of integrin in intracellular signaling. J Cell Sci 115: 2199–2206. 11973360 10.1242/jcs.115.10.2199

[75] Ohta Y, Hartwig JH, Stossel TP (2006) FilGAP, a Rho- and ROCK-regulated GAP for Rac binds filamin A to control actin remodelling. Nat Cell Biol 8: 803–814. 16862148 10.1038/ncb1437

[76] Nimnual AS, Taylor LJ, Bar-Sagi D (2003) Redox-dependent downregulation of Rho by Rac. Nat Cell Biol 5: 236–241. 12598902 10.1038/ncb938

[77] Huveneers S, Danen EH (2009) Adhesion signaling - crosstalk between integrins, Src and Rho. J Cell Sci 122: 1059–1069. 19339545 10.1242/jcs.039446

[78] Cox EA, Bennin D, Doan AT, O'Toole T, Huttenlocher A (2003) RACK1 regulates integrin-mediated adhesion, protrusion, and chemotactic cell migration via its Src-binding site. Mol Biol Cell 14: 658–669. 12589061 10.1091/mbc.E02-03-0142 PMC149999

[79] Doan AT, Huttenlocher A (2007) RACK1 regulates Src activity and modulates paxillin dynamics during cell migration. Exp Cell Res 313: 2667–2679. 17574549 10.1016/j.yexcr.2007.05.013 PMC2679865

[80] de Hoog CL, Foster LJ, Mann M (2004) RNA and RNA binding proteins participate in early stages of cell spreading through spreading initiation centers. Cell 117: 649–662. 15163412 10.1016/s0092-8674(04)00456-8

